# Retraction: Survival Analysis of Adult Tuberculosis Disease

**DOI:** 10.1371/journal.pone.0204676

**Published:** 2018-09-20

**Authors:** The PLOS ONE Editors

Following publication of this article, concerns were raised about the survival analysis: there are inaccuracies in the number of bacteriologically confirmed tuberculosis patients, patients assessed multiple times were overrepresented in the dataset, and the statistical analysis to handle risk factors with more than two categories is incorrect. In addition, there are concerns that the corresponding author completed the submission of the manuscript without approval by the listed co-authors.

The University College Cork has completed an investigation and confirmed the concerns raised.

In light of the concerns about the methodology and conclusions reported in the article and in line with the recommendation by the University College Cork, the *PLOS ONE* Editors retract this publication.

Zubair Kabair agreed with the retraction. Olurotimi Bankole Ajagbe and Terry O’Connor did not respond to the notification for retraction.
